# Genome editing of *CXCR4* by CRISPR/cas9 confers cells resistant to HIV-1 infection

**DOI:** 10.1038/srep15577

**Published:** 2015-10-20

**Authors:** Panpan Hou, Shuliang Chen, Shilei Wang, Xiao Yu, Yu Chen, Meng Jiang, Ke Zhuang, Wenzhe Ho, Wei Hou, Jian Huang, Deyin Guo

**Affiliations:** 1 School of Basic Medical Sciences, Wuhan University, Wuhan, 430071, PR China; 2 College of Life Sciences, Wuhan University, Wuhan, 430072, PR China; 3 Renmin Hospital of Wuhan University, Wuhan 430060, PR China; 4 The Center for Animal Experiment and ABSL-3 Laboratory, Wuhan University School of Medicine, Wuhan, 430071, PR China; 5 Department of Pathology and Laboratory Medicine, School of Medicine, Temple University, Philadelphia, PA 19103.

## Abstract

Genome editing via CRISPR/Cas9 has become an efficient and reliable way to make precise, targeted changes to the genome of living cells. CXCR4 is a co-receptor for the human immunodeficiency virus type 1 (HIV-1) infection and has been considered as an important therapeutic target for AIDS. CXCR4 mediates viral entry into human CD4^+^ cells by binding to envelope protein, gp120. Here, we show that human CXCR4 gene is efficiently disrupted by CRISPR/Cas9-mediated genome editing, leading to HIV-1 resistance of human primary CD4^+^ T cells. We also show that the Cas9-mediated ablation of CXCR4 demonstrated high specificity and negligible off-target effects without affecting cell division and propagation. The precise and efficient genome editing of CXCR4 will provide a new strategy for therapeutic application against HIV-1 infection.

The human immunodeficiency virus (HIV-1) has caused a global epidemic since it was found and confirmed to be the pathogen of acquired immunodeficiency syndrome (AIDS) in 1983. It can cause progressive immunodeficiency and severe neurocognitive disorders and eventually lead to fatal AIDS^1^. Although the highly active antiretroviral therapy (HAART) can reduce viremia to clinically undetectable levels and extend the lives of HIV-1 infected individuals^2^,^3^,^4^, it has many limitations such as high cost and side effects such as drug resistance and toxicity^5^. Moreover, reservoir of latent HIV-1 infection can cause a virus rebound once the antiretroviral therapy (ART) is discontinued^6^. Hence, there is an urgent need to develop alternative therapeutic approaches. The HIV-1 entry is mediated by its surface envelope glycoprotein by sequential binding to cellular primary receptor CD4^7^ and then a chemokine receptor CCR5 (R5-tropic)^8^ or CXCR4 (X4-tropic)^9^. The CCR5, which is expressed in lymphocytes, myeloid cells or CD4^+^ T cell subsets, is responsible for establishment of new infections and is dominant in the chronic phase of infection. The rare individuals of naturally occurring homozygous *CCR5-Δ32* mutation are highly resistant to HIV-1 infection and have no obvious phenotype changes except for increasing susceptibility to some pathogens^10^,^11^. Once infection is established, HIV-1 can use CXCR4 as an alternative receptor for entry. The X4-tropic HIV-1 strains are present in half of late-stage infections and are associated with more rapid disease progression^12^. Based on previous findings, both CCR5 and CXCR4 can serve as therapeutic targets by genome engineering technologies.

The naturally occurring homozygous *CCR5-Δ32* mutation confers resistance to HIV infection after transplantation with *CCR5Δ32/Δ32* stem cells^13^. Moreover, it has been shown that disruption of CCR5 receptor of autologous CD4^+^ T cells by zinc finger nucleases (ZFNs) can efficiently inhibit HIV-1 infection in CD4^+^ T cells^14^. In addition, genetic modification of both *CCR5* and *CXCR4* in primary human CD4^+^ T cells by ZFN protects cells from infection of CCR5 and CXCR4 trophic HIV-1 strains^15^. Recently, genetic perturbation mediated by the clustered regularly interspaced short palindromic repeat (CRISPR)-CRISPR-associated protein 9 (Cas9) provides an alternative approach for gene disruption and genome editing.

The CRISPR-Cas system was originally identified in bacteria and archaea as part of an adaptive immune system, consisting of CRISPR RNAs (crRNAs) and CRISPR-associated proteins to recognize and degrade complimentary sequences of invading virus and plasmids^16^. This system has been shown to have enormous potential for gene editing in a variety of hosts such as plants, zebrafish, drosophila, mice, rhesus and also in human cells^16^,^17^,^18^. The state-of-the-art genome editing tool of Type II CRISPR/Cas9 system induces DNA double strand breaks (DSBs)^19^. The DSBs can stimulate cell repair mechanisms including non-homologous end joining (NHEJ) and homology-directed repair (HR), but in most circumstances, NHEJ is the predominant mechanism for repairing DSBs^20^,^21^. This repair pathway is attended with nucleotide insertions, deletions or frame-shift mutations, consequently leading to gene disruption or modifications^22^.

Recently, *CCR5* has been successfully targeted using transcription activator like effector nucleases (TALEN) and CRISPR/Cas9 in pluripotent stem cells and hematopoietic stem cells^23^,^24^. However, targeting *CXCR4* by CRISPR/Cas9 remains to be developed. In the current study, we used the CRISPR/Cas9 system to introduce CXCR4 loss-of function mutations in Ghost-CXCR4 cells, Jurkat cells and primary human CD4^+^ T cells. The biallelic inactivation of CXCR4 by lentivirus-mediated delivery of CRISPR/cas9 constructs rendered the modified cells resistant to HIV-1 infection. Sequence analysis of predicted off-target sites revealed specific targeting of *CXCR4* and negligible off-target mutagenesis. Therefore, CRISPR/Cas9 disruption of *CXCR4* provides an excellent gene modification tool for therapeutic application in the future.

## Results

### CRISPR/Cas9-mediated genome editing of *CXCR4* protects Ghost X4 cells from HIV-1 infection

**Figure 1:**
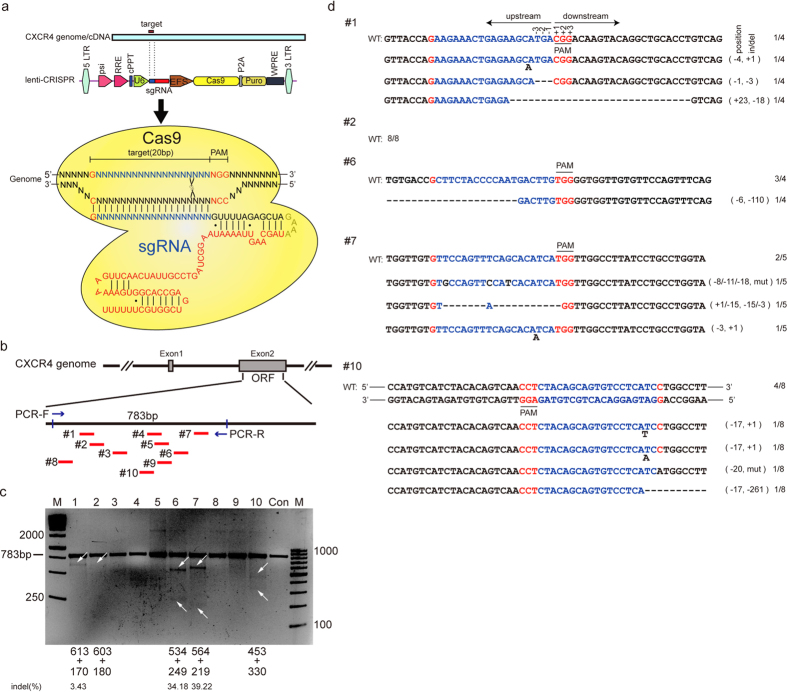
LentiCRISPR/Cas9 mediated editing of the human CXCR4 gene in Ghost cells. (**a**) Schematic diagram of gRNAs targeting the CXCR4 locus. RNA-guided gene targeting in eukaryotic cells involves the expression of the Cas9 protein and a gRNA under the control of the human U6 polymerase III promoter. Cas9 unwinds the DNA duplex and cleaves both strands upon recognition of a target sequence by the gRNA when the 3′ end of the target contains a protospacer adjacent motif (PAM) domain. Any genomic sequence of the 5′-GN_19_NGG-3′ or 5′-CCNN_19_C-3′ form can, in principle, be targeted. For all gRNAs, the initial 5′G (red) can improve transcription. (**b**) Schematic of the gRNAs for targeting *CXCR4*. gRNAs are shown in red. Blue arrows represent the primer pair used to amplify the region. (**c**) T7EN1 assay of each gRNA-lentiCRISPR/Cas9 targeting CXCR4 in Ghost cells. The Ghost X4 cells were infected with specified CXCR4-gRNA lentivirus at an M.O.I. of 40. The genomic DNA from the cells was extracted and PCR amplified to test for CXCR4 disruption by T7 endonuclease I assay using a 1.5% agarose gel. The lower migrating bands (indicated by arrows) in lanes indicate the disrupted CXCR4 alleles. Percentage of indels were measured by Image J software. (**d**) DNA sequences of CXCR4 loci of the modified Ghost cells. The total PCR products (c, upper band, about 783bp) were inserted into T-vector and analyzed by DNA sequencing. The PAM sequences are lined and highlighted in red; the targeting sequences were shown in blue; the mutations in target sequences in black; deletions are indicated with(-) and insertions with (+). N/N indicates ratio of WT or mutations to total sequenced clones. No mutations in control samples were observed.

In order to genetically disrupt the *CXCR4* allele, we designed 10 gRNAs to target Cas9 to the conserved sites of human and Rhesus macaque *CXCR4* gene (Fig. 1a) and generated a modular lentiviral sgRNA:Cas9 vector to deliver gRNAs into cells. To test the efficiency of each gRNA to direct Cas9-mediated ablation of CXCR4, we infected Ghost X4 cell line which is derived from the human osteon sarcoma (HOS) cells expressing CXCR4^25^,^26^ with the lentivirus at a multiplicity of infection (M.O.I.) of 40. Three days after the transduction, we performed T7EN1 assays to measure the insertion/deletions (indels) at CXCR4 locus and observed that CXCR4 was abrogated in up to 39.22% of Ghost cells depending upon the gRNA used (Fig. 1b,c). Upon Sanger sequencing, we observed that four out of ten gRNAs efficiently induced indel mutations in CXCR4 gene (Fig. 1d).

**Figure 2:**
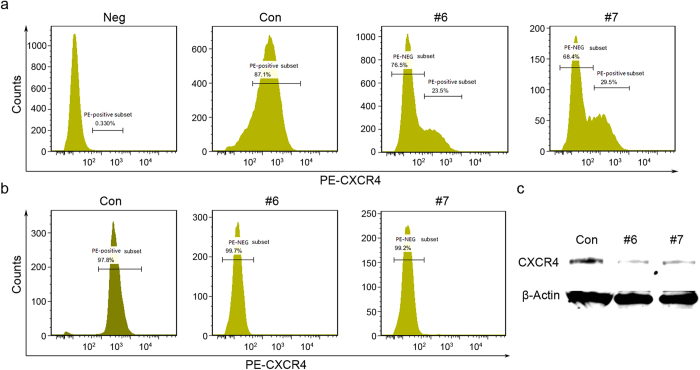
LentiCRISPR/Cas9 mediated disruption of surface CXCR4 in Ghost cells. (**a**) Flow cytometry analysis of CXCR4 expression in lentiCRISPR/Cas9-transduced Ghost X4 cells. The Ghost X4 cells transduced with CXCR4-gRNA lentivirus (M.O.I. of 40) were stained with PE-conjugated antibody specific for CXCR4 and analyzed by flow cytometry. The CXCR4-gRNA lentivirus treatment is marked on the top of each panel. For CXCR4-gRNA/Cas9 edited Ghost X4 cells, there was a distinction between specific CXCR4 positive and negative populations. The values shown are the percentages of CXCR4 positive or negative cells. The unstained Ghost X4 cells was considered as a negative control and the cells infected with lenti-CRISPR virus as a positive control. Analysis was done in Flow Jo (Tree star) software. (**b**) Purified population of CXCR4-gRNA lentivirus-transduced Ghost X4 cells generated in Fig. 1a, which were sorted by FACS for the lack of cell surface CXCR4. (**c**) Western blot analysis of CXCR4 expression in the Ghost X4 cells transduced with CXCR4-gRNA lentivirus (as in a). The lentivirus-treated Ghost cells were collected and analyzed by Western blotting with an anti-CXCR4 or anti-β-Actin antibody.

To further evaluate the efficiency of gRNA-mediated CXCR4 disruption in Ghost X4 cells, we performed flow cytometry assay to analyze CXCR4 protein levels 48 hours post transduction. We found that the number of CXCR4-expressing cells reduced to 23.5% and 29.5% of the population after treatment with gRNA #6 or #7 respectively (Fig. 2a). The FACS-sorted cells showed homogeneity in lack of CXCR4 expression (Fig. 2b). A fraction of transduced cells from Fig. 2a were collected and subjected to western blots and the results showed that the expression level of CXCR4 was remarkably reduced after transduction with CXCR4-gRNA lentivitus (Fig. 2c).

**Figure 3:**
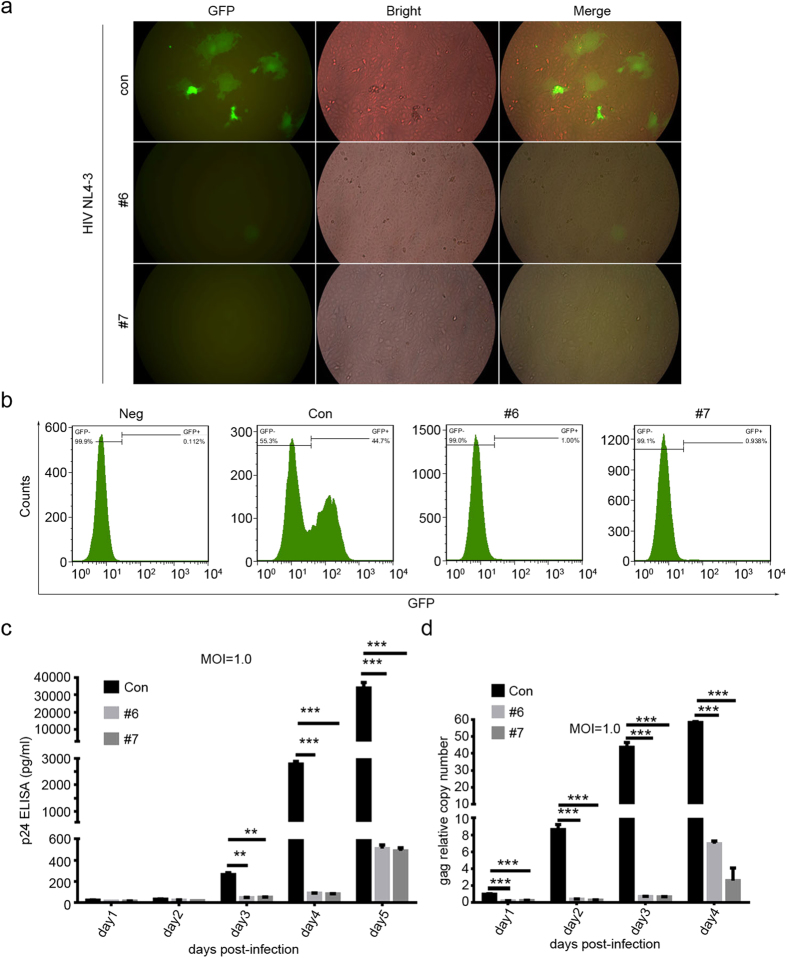
CXCR4 disruption by CRISPR/Cas9 protects Ghost X4 cells from HIV-1 infection. (**a**) Fluorescence microscopy detection of GFP expression activated by HIV-1 infection. Unmodified or CXCR4-gRNA/Cas9-modified Ghost X4 cells sorted by FACS were exposed to HIV-1_NL4-3_ at an M.O.I. of 0.1. After 6 hours infection, the cells were washed three times with PBS and cultured in fresh medium. Fluorescence microscopy was used to detect HIV-1 infection after three days of virus exposure. The Ghost X4 cells are stably transduced with MV7neo-T4 (CD4) retroviral vector and contain a stable element of LTR-GFP reporter. Upon HIV-1 infection, the Tat protein will bind to the LTR promoter to further activate the GFP expression. (**b**) FACS analysis of EGFP expression activated by HIV-1 infection. Assays performed as in (**a**) except for the HIV-1_NL4-3_ infection at a M.O.I. of 1.0. The percentage of HIV-1-infected Ghost X4 cells was quantified by determining GFP-positive cells using FACS four days post-infection. (**c**,**d**) p24 ELISA and qPCR detection of HIV-1_NL4-3_ level in unmodified or CXCR4-gRNA/Cas9-modified Ghost X4 cells sorted by FACS. HIV-1 infection as performed in (**b**). At the indicated time points of post-infection, the culture supernatants were harvested for ELISA detection of HIV-1 p24 expression while the genomic DNA of the cells was extracted and subjected to quantitative real-time PCR using gene-specific primers for HIV-1 gag and human β-globin gene. In (**d**), the relative copy numbers of gag were normalized to β-globin. Normalization was carried out based on gag gene amplification in non-modified cells with HIV-1 infection at the time of 1 day. Mean ± SEM represents the average value of three independent experiments. *p < 0.05, **p <0.01, ***p < 0.001.

We next tested whether CRISPR/Cas9-mediated disruption of *CXCR4* gene could inhibit HIV-1 infection by taking advantage of the Ghost X4 reporter cell line containing green fluorescent protein (GFP) expression cassette driven by the HIV-1 LTR element, which permits the evaluation and quantitation of HIV-1 infection by flow cytometry. We transduced Ghost X4 reporter cells with lentivirus carrying gRNA #6 or #7, stained cells with anti-CXCR4-PE and sorted cells that were deficient in CXCR4 expression. We next infected the sorted Ghost X4 cells (as in Fig. 2b) with HIV-1_NL4-3_, a CXCR4-tropic HIV-1 virus at M.O.I of 1.0 and measured their GFP levels by a fluorescence microscope. We show that Ghost X4 cells modified with #6 or #7 gRNA/Cas9 lentivirus were negative for GFP expression compared to control cells (Fig. 3a), indicating that CRISPR/Cas9-mediated genome editing of CXCR4 protects Ghost X4 cells from HIV-1 infection. To quantitatively test whether alteration of CXCR4 could confer protection to Ghost X4 cells, we analyzed the percentage of GFP positive cells by flow cytometry 4 days after viral infection. We observed the complete loss of GFP expression in the CXCR4-edited cells compared to control cells which contain 55.3% GFP positive cells (Fig. 3b), suggesting a limited HIV-1 infection in the cells with modified CXCR4. Furthermore, we performed p24 antigen ELISA (Fig. 3c) and real-time PCR analysis (Fig. 3d) and found that the virus was undetectable until day 3 and the virus titer and copy number of HIV-1_NL4-3_ decreased significantly at day 4 or day 5 in the genome-edited Ghost X4 cells compared to control cells. Taken together, these results demonstrated that both #6 and #7 gRNA mediated Cas9 cleavage of CXCR4 can protect Ghost X4 cells against HIV-1 infection.

### CXCR4 gene disruption by CRISPR/Cas9 confers Jurkat T cells resistant to HIV-1 infection

**Figure 4:**
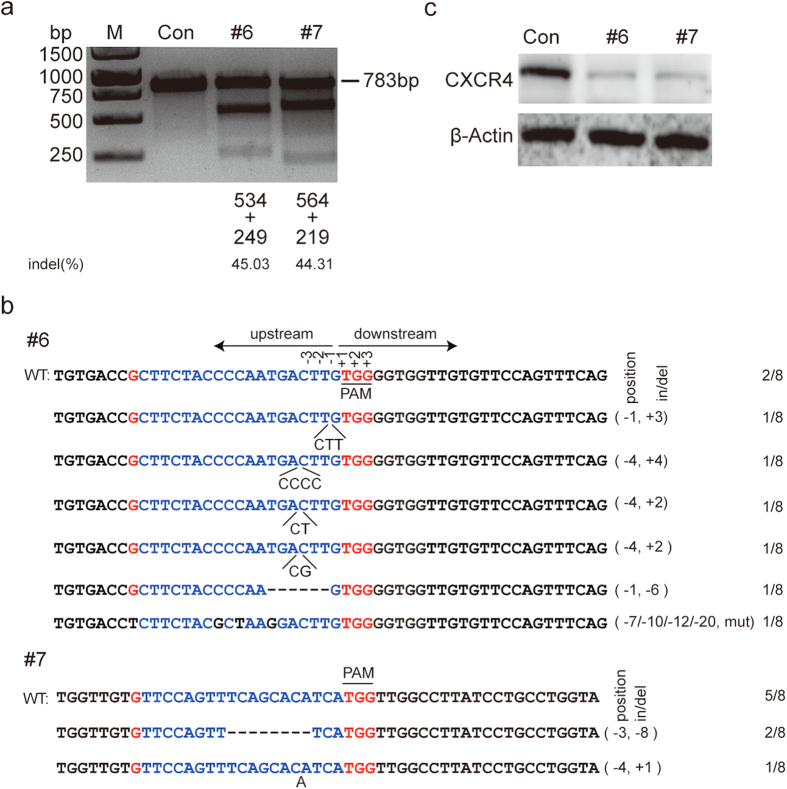
CXCR4 deletion efficacy by selected gRNA/Cas9 in Jurkat T cells. (**a**) CXCR4 genomic disruption was detected by T7EN1 assay. Jurkat T cells were transduced with control or CXCR4-gRNA lentivirus at an M.O.I. of 40. After 48 hours infection, the genomic DNA was extracted and PCR amplified for T7EN1 assay. The expected DNA length of wildtype allele was 783 bp. The lower bands indicated the disrupted CXCR4 alleles. Percentage of indels were measured by Image J software. (**b**) DNA sequences of CXCR4 loci of the modified Jurkat cells. Assays were performed as in Fig. 1c,d. (**c**) Western blot analysis of CXCR4 expression in the CXCR4-gRNA lentivirus transduced Jurkat T cells. Jurkat T cells were transduced with control or CXCR4-gRNA lentivirus at an M.O.I. of 40. After 48 hours, cells were collected and analyzed by Western blotting with an anti-CXCR4 or anti-β-actin antibody.

Human CD4^+^ T cells are the major target cells of HIV-1 infection. To determine whether *CXCR4* gene disruption by CRISPR/Cas9 can protect Jurkat T cells from HIV-1 infection, we transduced the Jurkat T cells with the control, #6 or #7 CXCR4-gRNA/Cas9 lentivirus and performed T7EN1 assay. As shown in Fig. 4a, we observed high on-target efficiency for #6 and #7 gRNA with 45.03% and 44.31% ablation of *CXCR4* gene, respectively. Sanger DNA sequencing of the targets confirmed that the mutations were introduced in CXCR4 gene in Jurkat T cells transduced with #6 or #7 gRNA/Cas9 lentivirus (Fig. 4b). In addition, we also collected some cells after transduction for western blot analysis and observed a significant decrease of CXCR4 protein expression in cells that were transduced with #6 or #7 gRNA/Cas9 (Fig. 4c).

**Figure 5:**
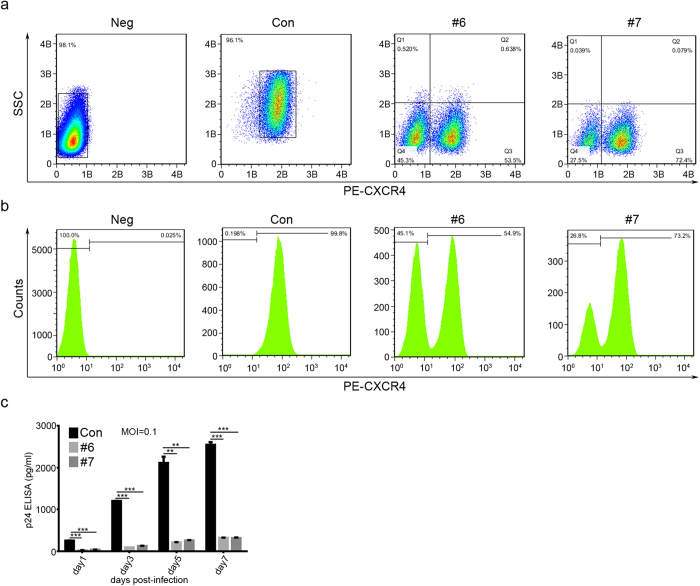
Jurkat T cells treated with CXCR4-gRNA lentivirus survived from HIV-1 challenge. (**a**,**b**) FACS analysis showing the lack of CXCR4 expression in CXCR4-gRNA lentivirus-treated Jurkat T cells. The lentivirus-transduced Jurkat T cells were stained with PE-CXCR4 antibody and analyzed by FACS (as in Fig. 2). Treatment was noted on the top of each panel. The unstained Jurkat T cells were determined as a CXCR4 negative subset while the control (empty vector) treated cells as a CXCR4 positive subset. The transduced Jurkat T cells expressing CXCR4-gRNA (#6 and #7) revealed two cell subsets (CXCR4-negative and positive). (**c**) Jurkat T cells treated with CXCR4-gRNA were highly resistant to HIV-1 challenge. Unmodified or CXCR4-gRNA/Cas9-modified Jurkat T cells were infected with HIV-1_NL4-3_ at an M.O.I. of 0.1. At the different time points, the cell-free supernatant was harvested and titrated with p24 capture ELISA. The graph represents one of three independent infection experiments and error bars represent SEM. *p < 0.05, **p < 0.01.

To evaluate CXCR4 protein expression in Jurkat T cells after lenti-CXCR4-gRNA/Cas9 transduction with the control, #6 or #7 CXCR4-gRNA/Cas9 lentivirus, we stained cells with anti-CXCR4-PE and performed flow cytometry analysis. We found that cells transduced with #6 or #7 gRNA displayed two subsets of cells that represent CXCR4-negtive and -positive populations (Fig. 5a,b). In contrast, the negative (unstained Ghost X4 cells) or positive control (mock lentivirus) cells showed only one subset of cells (either CXCR4-negtive or -positive cells) (Fig. 5a,b). To test whether alteration of CXCR4 can confer protection to HIV-1_NL4-3_ virus, we infected modified Jurkat T cells with HIV-1_NL4-3_ virus and performed p24 antigen ELISA assays at 1, 3, 5 and 7 days post infection. We observed that the p24 level of HIV-1_NL4-3_ was much lower in genome-edited Jurkat T cells compared to the control (Fig. 5c). All these data demonstrate that the #6 or #7 gRNA/Cas9 can disrupt CXCR4 gene in Jurkat T cells, resulting in the protection against HIV-1 infection.

### CXCR4-modification of human primary CD4^+^ T cells by CRISPR/Cas9 confers cell resistance to HIV-1 infection

**Figure 6:**
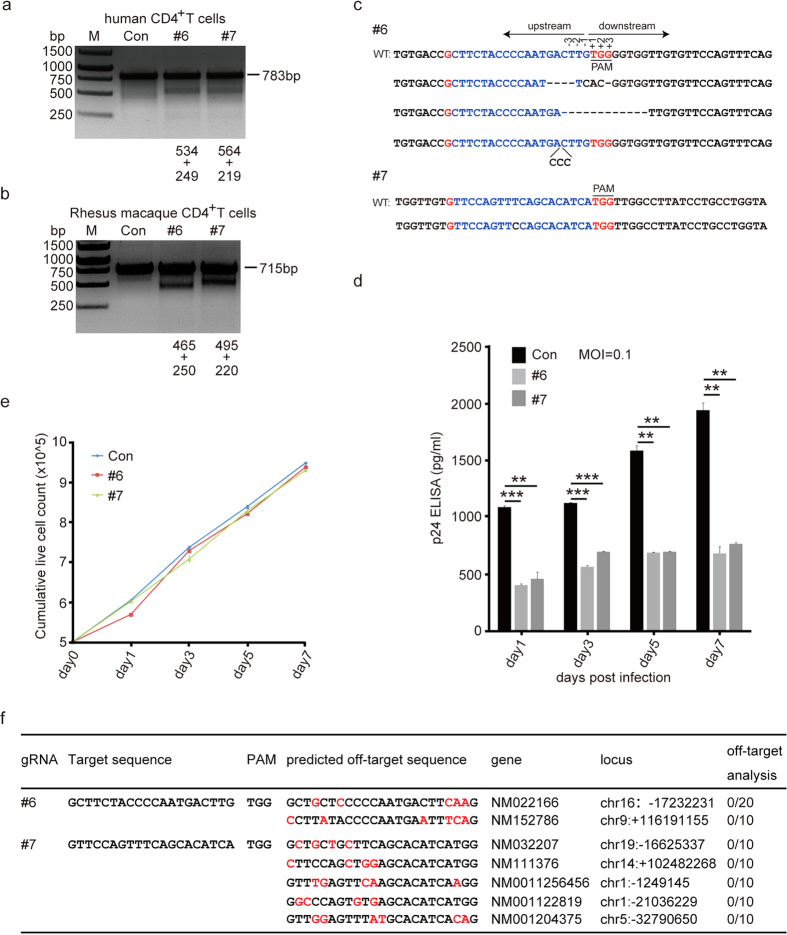
LentiCRISPR/Cas9 disruption of CXCR4 in primary CD4^+^ T cells leads to inhibition of HIV-1 infection. (**a**) LentiCRISPR/Cas9 editing of CXCR4 in CD4^+^ T cells. Primary human CD4^+^ T cells were transfected with CXCR4-gRNA/Cas9 or control plasmids. After 36 hours, the cells were collected for genomic DNA extraction and T7EN1 assay. The lower bands in lane 6 and 7 indicate mutant CXCR4 alleles. (**b**) LentiCRISPR/Cas9 efficiently disrupts CXCR4 in Rhesus macaque CD4^+^ T cells. The #6 and #7 CXCR4-gRNA target sites are conserved between humans and Rhesus macaques. Disruption frequencies were measured by the T7EN1 assay. (**c**) Sequences of modified CXCR4 gene in the human CD4^+^ T cells. The PCR products (in a) were cloned into T-vector and 10 recombinants were analyzed by DNA sequencing. No mutations were observed in control samples. (**d**) The modified human CD4^+^ T cells resist HIV-1 infection. Primary CD4^+^ T cells (2 × 10^6^) in a 6-well plate were stimulated and transfected with control, #6 or #7 CXCR4-gRNA/Cas9 plasmids by Amaxa 4D-Nucleofector. After 48 hours, the HIV-1_NL4-3_ virus (M.O.I of 0.1) was added to the cells for 4 hours infection, and then the cell supernatants were removed and fresh medium was added. At the indicated time points, the cell-free supernatants were collected and tested by p24 capture ELISA to determine the viral titers. The graph represents one of 3 independent infection experiments and error bars represent SEM. *p <0.05,  **p <0.01,  ***p < 0.001. (**e**) Assessment of cell propagation with loss of CXCR4 by CRISPR/Cas9. Primary human CD4^+^ T cells were transfected and the total live cells were counted at different times after transfection. Data is from one of 3 independent experiments. (**f**) Mutation frequency analysis at the predicted off-target sites. The off-target sites were predicted and aligned with human genome. The lentiCXCR4-gRNA/Cas9 virus infected Ghost X4 cells were collected for genomic DNA extraction and PCR amplified for the predicted off-target sites. The PCR products were then cloned into T-vector and subjected to sequencing. The off-target sequences in red color indicate different bases from on-target sites. At least 10 recombinant clones were sequenced and no off-target effects were observed. N/N indicates the number of mutations to total sequenced colonies.

Given the high efficiency of genome editing of CXCR4 by lentiCRISPR/Cas9 in Ghost X4 and Jurkat T cells, we tested the feasibility of deleting CXCR4 in human primary CD4^+^ T cells. We transfected the activated primary human CD4^+^ T cells with control, #6 or #7 CXCR4-gRNA/Cas9 plasmids and performed T7EN1 assays 3 days post transfection. We observed that both #6 and #7 gRNA induced indel mutations in CXCR4 gene in primary T cells (Fig. 6a), which was less efficient than Jurkat cells (Fig. 5a). DNA sequencing analysis of 10 clones confirmed indels in the #6 or #7 gRNA target sites of CXCR4 gene in CD4^+^ T cells (Fig. 6c). To determine the efficiency of CXCR4-gRNA/Cas9 modification in providing protection to primary CD4^+^ T cells against HIV-1 infection, we infected the cells (containing modified CXCR4) with CXCR4-tropic HIV-1_NL4-3_ and cultured for 7 days. ELISA assays showed that the p24 level decreased by over 64% in cells transfected with #6 or #7 CXCR4-gRNA/Cas9 compared to the control cells at day 7 (Fig. 6d). These results demonstrate that disruption of CXCR4 expression in human CD4^+^ T cells by CRISPR/Cas9 confers partial protection to HIV-1 infection.

It has been well documented that CXCR4 plays critical roles in hematopoietic cells, thymic differentiation as well as progenitor cell migration and homing^27^,^28^. In addition, the potential off-target effects of CRISPR/Cas9 can induce gene toxicity in cells and increase the risk of cell death^19^,^29^, and we then investigated whether the loss of CXCR4 could affect cell viability. We transfected human primary CD4^+^ T cells with control, #6 or #7 CXCR4-gRNA/Cas9 and counted the live cells for 7 days. As shown in Fig. 6e, we observed that the growth rate and proliferation of cells transfected with #6 or #7 were similar to that of the control plasmid transfected cells.

### CXCR4 allele disruption mediated by lentiCRISPR/Cas9 in Rhesus macaque CD4^+^ T cells

Rhesus macaque is an excellent non-human primate model to test HIV-1 gene therapy methods and measure the safety and efficacy of anti-viral vaccines. In order to provide a proof of concept for future clinical therapy studies using the lentiCRISPR/Cas9 system, we isolated CD4^+^ T cells from the whole blood of Rhesus macaque and stimulated it with anti-CD3/anti-CD28 coated beads for transfection. As the target sites of CXCR4 we selected for this study are identical in Rhesus and humans, we transfected Rhesus CD4^+^ T cells with #6 or #7 gRNAs and performed T7EN1 assay. Our results show that both #6 and #7 gRNA induced CXCR4 gene mutation in Rhesus macaque CD4^+^ cells (Fig. 6b), indicating that CXCR4 genome editing can be achieved by lentiCRISPR/Cas9 in Rhesus macaque CD4^+^ T cells.

### Off-target analysis indicates high specificity of lentiCXCR4-gRNA/Cas9-mediated gene disruption

The CRISPR/Cas9 system represents a powerful tool for genome engineering; however, the potential off-target cleavage is always a serious concern for future therapeutic applications^30^. In order to determine the off-target effects of the lentiCXCR4-gRNA/Cas9, we aligned the #6 and #7 target sequences to the human genome to search for all the potential off-target sites using an online tool (http://crispr.mit.edu)^19^. As shown in Fig. 6f, we identified two and five potential off-target sequences with high scores for #6 and #7 gRNA, respectively. Next, we PCR amplified genomic DNA after transduction and sequenced the 500-bp DNA spanning the off-target region using TA cloning. As shown in Fig. 6f, we did not detect any mutations at these sites in lentiCRISPR/Cas9 transduced Ghost X4 cells, suggesting that there is no off-target mutagenesis in our single gRNA transfection experiment.

## Discussion

HAART is being used effectively to combat HIV, but it cannot eradicate the virus. Interestingly, the *CCR5 delta32* homozygous allogeneic bone marrow transplant to HIV infected patients successfully eradicated HIV to an undetectable level^31^. However, broad clinical application of this approach is restricted due to toxicity by allogeneic rejection and very limited availability of HLA-matched *CCR5 delta32* homozygous donors. Recently, ZFN mediated genetic modification in autologous CD4^+^ T cells has proven to be effective in disruption of *CCR5* and may become an alternative therapy to treat AIDS^14^. *CCR5* can also be efficiently disrupted in hematopoietic stem cells (HSCs) to inhibit HIV challenge in humanized mice^32^,^33^. In addition, the gene modified autologous HSCs can be transplanted into HIV-infected individuals^34^. These strategies are only directed against CCR5 trophic viruses. However, previous reports showed that 46% of ART-treated individuals harbor both R5 and X4 strains and 4% with only X4-HIV strains, and that the CCR5 virus can evolve to use CXCR4 as co-receptor to infect cells^35^. Therefore, disruption of *CXCR4* could be a complementary strategy for inhibiting HIV-1 infection, especially in patients with chronic disease progression^15^,^36^,^37^. In order to achieve complete clearance of HIV, simultaneous disruption of both *CCR5* and *CXCR4* in primary human CD4^+^ T cells may be required for treatment of established HIV-1 infection^15^. ZFNs were also designed to target proviral DNA that is inserted into the human genome^38^ and disrupt *CXCR4* in human CD4^+^ T cells^36^. Although, ZFNs can serve as a powerful technology to edit genome, the desired DNA binding specificity requires an extensive screening process and the modular construction is labor intensive and costly. Recently, the CRISPR/Cas9 system has been developed to manipulate the genome and its ability to precisely modify endogenous genes can facilitate disease treatment. In this study, we applied CRISPR/Cas9 system to target *CXCR4* gene and identified two efficient target sites. The two gRNAs can mediate Cas9 disruption of *CXCR4* in Ghost-X4 cells, Jurkat cells as well as primary human CD4^+^ T cells with an efficacy of more than 40% mutagenesis. Furthermore, we also show that these gene modified cells were resistant to infection by X4 tropic HIV-1. Thus, the CRISPR/Cas9-mediated deletion of *CXCR4* provides an alternative way to treat X4 tropic HIV infection.

However, for therapeutic purposes, the nuclease specificity of Cas9 is an important factor to be considered^39^. Previous studies showed that among five gRNA designed to target human *CCR5* gene by CRISPR/Cas9 system, two of them have potential off-target activity at the human C-C chemokine receptor type 2 (CCR2) gene^40^. In another report, a careful BLAST-search in the NCBI database showed that a 20-nt sequence of gRNA against the human *CCR5* gene showed no potential off-target effects in induced pluripotent stem cells (iPSCs)^41^. Recent studies showed that the CRISPR/Cas9 editing of CD34^+^ HSCs with single gRNA induced highly efficient CCR5 ablation and low off-target mutagenesis^23^. Furthermore, the lentivirus^42^ and adenovirus- induced^43^ Cas9 knockout of CCR5 were also reported to have low off-target effects. In our studies, the off-target sequences were predicted for each CXCR4 gRNA using optimized CRISPR design tool as described previously^44^. The potential sequences were further aligned to the human genome to search for effective off-target sites. We did not detect any off-target modifications as analyzed by Sanger sequencing. Therefore, in this study we show that the CRISPR/Cas9 system proves to be specific and highly efficient for targeting *CXCR4*.

It is of concern that the ablation of *CXCR4* may impair hematopoietic cells and thymic differentiation as well as progenitor cell migration and homing^27^,^28^. The CXCR4 and its ligand CXC12 (SDF-1) play important roles in retention of HSCs in the bone marrow, and loss of CXCR4 expression in zygote leads to embryonic lethality of mice^45^. Therefore, it may not be feasible to completely knock out CXCR4 in clinical trials. However, ZFN disruption of *CXCR4* has no effect on human primary CD4^+^ T cell proliferation^36^,^37^. In accordance with previous reports, our results also revealed that disruption of CXCR4 by CRISPR/Cas9 did not lead to a decrease in cell growth. In addition,, Cxcr4-deficient human T cells still retain their immune function (at least partially) in a mouse model^36^,^46^. Therefore, one possibility for knocking out CXCR4 in human therapy may be the temporal ablation of CXCR4 for mature post-thymic CD4^+^ T cells. As there are no naturally occurring homozygous CXCR4 mutations found in humans, the consequences of knocking out CXCR4 in specific cell types should be further studied and explored before it can be used for clinical therapy. In addition, our previous work showed that the CXCR4 mutant, P191A, can replace endogenous CXCR4 to maintain the normal function but reduce HIV-1 replication^29^. Hence, in future studies involving HIV-1 gene therapy, we propose that a physiologically functional CXCR4 (e.g. mutant P191A) can be introduced when CXCR4 of the HSCs is disrupted so as to generate HIV-1 resistance and at the same time to reconstitute the physiological function of CXCR4.

In summary, we found two sites in *CXCR4* that can be targeted effectively and specifically by the CRISPR/Cas9 system, resulting in co-receptor *CXCR4* ablation. We also show that the modified cells are resistant to X4 type HIV-1 infection and this may provide us with an alternative approach of gene therapy for treating AIDS. Although lenti-CRISPR/Cas9 provides powerful means to disrupt *CXCR4*, the optimized delivery methods using adenovirus need to be explored as to further improve their specificity and minimize the concern for therapeutic safety. Due to the variation in viral infection, co-disruption of *CCR5* and *CXCR4* should be tested using lenti- or adenovirus mediated CRISPR/Cas9 system in the future. Furthermore, the successful disruption of *CXCR4* in Rhesus macaque CD4^+^ T cells may accelerate gene therapy studies for AIDS in non-human primate models.

## Methods

### Lenti CXCR4-gRNA-Cas9 constructs

The gRNA-coding cDNAs for ten targets conserved in both human and Rhesus monkey CXCR4 gene were designed and synthesized to make the lenti CXCR4-gRNA-Cas9 constructs as described previously^19^. Briefly, the 24-bp forward and reverse primers including 20 bp target sequence and *Bsm*bI sticky end were annealed and inserted into the lentiCRISPR-v2 plasmid (Addgene 52961) digested with *Bsm*bI (Fermentas). Primer sequences are shown in Table S1.

### Cell lines and isolation of primary CD4^+^ T cells

HEK293T cells, Ghost-CXCR4 (X4) cells, Jurkat T cells and HIV-1 virus were used as described previously^47^. Whole blood samples from healthy donors were purchased from the Wuhan Blood Center (Wuhan, China). The human peripheral blood mononuclear cells (PBMCs) were separated from the whole blood by centrifugation with Ficoll-Paque Premium (BD). The primary human CD4^+^ T cells were further purified and enriched by the CD4^+^ T cell isolation Kit (Miltenyi Biotech) according to the manufacturer’s instructions and then maintained in complete RPMI medium supplemented with 10% FBS. Before transfection, the primary human CD4^+^ T cells were stimulated 2 to 3 days with anti-CD3/anti-CD28-coated plate in the presence of recombinant human interleukin-2 (20 IU/ml, Roche Applied Science). The PBMCs of whole blood from healthy Rhesus macaque (grown in the Center for Animal Experiment and ABSL-3 Laboratory, Wuhan University, China) were isolated by centrifugation with Ficoll-Paque Premium (BD). The CD4^+^ T cells were further purified with a non-human primate CD4^+^ T cell selection kit (Miltenyi Biotech). The cells were then activated with anti-CD3 (clone FN-18)/anti-CD28 (clone L293) (Invitrogen) coated on culture plate for 3 days.

### Transfection of primary CD4^+^ T cells and cell counting

The stimulated primary human or Rhesus macaque CD4^+^ T cells were electro-transfected with CXCR4 gRNAs-Cas9 or control plasmids using the T cell Nucleofector kit #VPA-1002 (Amaxa Human T Cell Nucleofector Kit) or P3 Primary Cell 4D-Nucleofector × Kit (V4XP-3012). In brief, 5 × 10^6^ CD4^+^ T cells per sample were collected and washed twice in PBS. The cells were re-suspended in 100 μl Nucleofector Solution with 2 μg of plasmids respectively. The cell/DNA mixture was transferred into the certified cuvette and electro-transfected with an Amaxa Nucleofector II Device using the Nucleofector Program T-020 or a Lonza 4D-Nucleofector System. After transfection, the cells were cultured in RPMI 1640 medium supplemented with 10% FBS for two days and then used for further analysis. Cells were counted using trypan blue dye exclusion method on a hemocytometer under a microscope.

### Lentivirus and HIV-1 virus production and transduction

To produce lentivirus, the HEK 293T cells seeded in 100-mm plate were transfected with 6.0 μg lentiCXCR4-gRNA-Cas9 or lenti-CRISPR-v2 control plasmids, 4.5 μg psPAX2 and 3.0 μg VSV-G plasmids using Polyethylenimine reagent (PEI, Polysciences, Warrington, PA) according to the manufacturer’s instructions. After incubation for 72 hours, the supernatants of the transfected cells containing lentivirus were harvested and filtered with 0.45 μm filter and then stored at −80 °C. The viral titers were determined by virus counter (Virocyt 2100). The Ghost-CXCR4 cells or Jurkat T cells (1 × 10^5^) seeded in 12-well plate were transduced with the lentivirus at an m.o.i of 40. For the Jurkat T cells, the spin-transduction was performed by centrifuging the plate coated with 8 μg/ml polybrene (Sigma-Aldrich) at 1200 g for 2 hours at 25 °C and cultured for another 2 hours in the incubator. Then, the medium was changed with fresh RPMI 1640 supplemented with 10% FBS. These transduced cells were incubated for 2 days and then collected for genomic DNA extraction or stained with PE-CXCR4 antibody and evaluated by flow cytometry. The HIV-1 strain, NL4-3 (HIV-1_NL4-3_) was generated as described in our previous work^47^.

### T7EN1 assays for quantitating frequencies of indel (insertion or deletion) mutations and DNA sequencing

The genomic DNA was extracted from cells using a Blood & Cell Culture DNA Midi Kit (TIANGEN, China) according to the manufacturer’s protocol. To assess the mutation frequency, T7 endonuclease I assay (T7EN1) were performed as described previously^48^. Briefly, the genomic region flanking the gRNA target site was PCR amplified using primers listed in table S1. 800 ng purified PCR products were mixed with 2 μl NEB buffer 2.0 (New England Biolabs) and deionized H_2_O to make the total volume of 20 μl. The mixture was annealed to form heteroduplexes. After that, the heteroduplexes were digested with 1 μl T7 Endonuclease I (10 units/μl, New England Biolabs) at 37 °C for 30 minutes. The digested DNA was analyzed on electrophoresis system using a 1.5% agarose gel. The mutation frequency was quantified (Image J software, NIH Image-BioLab) and calculated as described previously^39^. For sequence analysis, the purified PCR product was cloned into the pGEM-T Easy vector (Promega) and then the recombinants were sequenced using a T7 primer.

### Flow cytometry analysis, Western blot and fluorescence microscopy

To determine HIV-1 infection efficiency, non-modified or CXCR4-gRNA/Cas9-modified Ghost X4 cells were exposed to HIV-1_NL4-3_ at an m.o.i of 1.0. After 6 hours, the cells were washed three times with PBS and cultured in fresh medium. At different time points, the cells were collected and assessed for GFP expression through flow cytometry in wash buffer (1 mM EDTA, 1.0% FBS, 1.0% formaldehyde, 0.2% glutaraldehyde in PBS). The results are presented as fluorescence histograms using GraphPad Prism 6.01 (GraphPad Software). To determine CXCR4 disruption efficiency, the lentivirus transduced Ghost X4 cells or Jurkat T cells were stained with PE (Phycoerythrin) conjugated anti-human CXCR4 antibody (Biolegend) and analyzed or sorted by flow cytometry (FACSan, Beckman Coulter). The data were plotted in FlowJo software (Treestar). A fraction of these transduced cells were lysed in a buffer (50 mM Tris-HCl pH 7.4, 150 mM NaCl, 1% NP-40, 0.25% deoxycholate) containing protease inhibitor cocktail (Roche Applied Science) for 30 min on ice. The lysates were denatured in 2× SDS loading buffer by boiling for 10 minutes and were subjected on a 4–15% continuous SDS-PAGE. After transferring the protein to nitrocellulose membranes (Millipore), the anti-CXCR4 antibody (ab2074, Abcam) and Immobilon Western chemiluminescent HRP substrate (Millipore) were used to detect cell surface CXCR4 expression. The bands were scanned by Fujifilm LAS 4000. For the fluorescence microscopy detection of GFP expression in Ghost X4 cells, the unmodified or CXCR4-gRNA/Cas9-modified cells were exposed to HIV-1_NL4-3_ at an m.o.i of 0.1. After 6 hours infection, the cells were washed three times with PBS and cultured in fresh medium. Fluorescence microscopy was used to detect HIV-1 infection after three days of virus exposure.

### Real time PCR and p24 antigen ELISA

6 × 10^4^ sorted Ghost-CXCR4 cells or unsorted Jurkat T cells were seeded in 12-well plate the day before HIV-1 infection. These cells were then challenged with HIV-1_NL4-3_ virus for 8 hours at an m.o.i. of 1.0 for Ghost-CXCR4 cells and 0.1 for Jurkat T cells in a total volume of 500 μl serum-free medium. The medium was removed and the cells were cultured with fresh complete medium after washing with PBS for 3 times. At different days post infection, the cell-free viral stock was detected for HIV-1 p24 antigen using a p24 antigen ELISA Kit (RETRO-TEK) and the cellular genomic DNA was isolated for quantitative PCR specific to HIV-1 gag or human β-globin gene using SYBR Green PCR Master Mix (Invitrogen). Primers are shown in Table S1.

### Off-target effect and statistical analysis

The CXCR4-gRNA sequences were blasted with online tool (http://crispr.mit.edu) to search for the predicted off-target sites. Six-bp mismatches compared with the target consensus sequence were allowed and the potential sequences were aligned to the human genome using web alignment tool (http://blast.ncbi.nlm.nih.gov). The sequences of these predicted off-target sites were PCR amplified and cloned into a pGEM-T vector for sequencing to identify the off-target activity. For statistical analysis, data were analyzed using OriginPro8.0 software. Statistical significance was determined with Student t test, with a *p* < 0.05 considered statistically significant. All experiments were performed for at least three times.

## Additional Information

**How to cite this article**: Hou, P. *et al.* Genome editing of *CXCR4* by CRISPR/cas9 confers cells resistant to HIV-1 infection. *Sci. Rep.* **5**, 15577; doi: 10.1038/srep15577 (2015).

## Supplementary Material

Supplementary Table S1
