# Supplementary material for: Genome editing of CXCR4 by CRISPR/cas9 confers cells resistant to HIV-1 infection
Source: Sci Rep. 2015 Oct 20;5:15577. doi: 10.1038/srep15577 (PMC4612538; doi:10.1038/srep15577)
Supplement: Supplementary Table S1 [file srep15577-s1.doc]

**Supplementary information for**

**Genome editing of *CXCR4* by CRISPR/cas9 confers cells resistant to HIV-1 infection**

Panpan Hou^1, 2^, Shuliang Chen^1^, Shilei Wang^2^, Xiao Yu^2^, Yu Chen^2^, Meng Jiang^3^, Ke Zhuang^4^, Wenzhe Ho^4^, Wei Hou^1^, Jian Huang^5^ and Deyin Guo^1^*

1. School of Basic Medical Sciences, Wuhan University, Wuhan, 430071, PR China

2. College of Life Sciences, Wuhan University, Wuhan, 430072, PR China

3. Renmin Hospital of Wuhan University, Wuhan 430060, PR China

4. The Center for Animal Experiment and ABSL-3 Laboratory, Wuhan University School of Medicine, Wuhan, 430071, PR China

5. Department of Pathology and Laboratory Medicine, School of Medicine, Temple University, Philadelphia, PA 19103.

*Corresponding author: Dr. Deyin Guo, E-mail: [dguo@whu.edu.cn](mailto:dguo@whu.edu.cn), School of Basic Medical Sciences, Wuhan University, Wuhan, 430072, PR China

**Running title: CRISPR/Cas9 editing of *CXCR4* inhibits HIV-1 infection**

**Table S1. The primers used in the study**

| **Constructs name** | **Primer sequence (5′-3′)** | **Annealing or PCR condition** |
| --- | --- | --- |
| LentiCRISPR CXCR4-1 | F:CACCGAAGAAACTGAGAAGCATGA  R:AAACTCATGCTTCTCAGTTTCTTC | 95 °C, 5 min; 95 °C to 85 °C, -2 °C/s; 85 to 25 °C, -0.1 °C/s |
| LentiCRISPR CXCR4-2 | F: CACCGAAGCATGACGGACAAGTAC  R :AAACGTACTTGTCCGTCATGCTTC | 95 °C, 5 min; 95 °C to 85 °C, -2 °C/s; 85 to 25 °C, -0.1 °C/s |
| LentiCRISPR CXCR4-3 | F :CACCGCCGTGGCAAACTGGTACTT  R :AAACAAGTACCAGTTTGCCACGGC | 95 °C, 5 min; 95 °C to 85 °C, -2 °C/s; 85 to 25 °C, -0.1 °C/s |
| LentiCRISPR CXCR4-4 | F:CACCGAAGCTGTTGGCTGAAAAGG  R:AAACCCTTTTCAGCCAACAGCTTC | 95 °C, 5 min; 95 °C to 85 °C, -2 °C/s; 85 to 25 °C, -0.1 °C/s |
| LentiCRISPR CXCR4-5 | F: CACCGCTGAAAAGGTGGTCTATGT  R :AAACACATAGACCACCTTTTCAGC | 95 °C, 5 min; 95 °C to 85 °C, -2 °C/s; 85 to 25 °C, -0.1 °C/s |
| LentiCRISPR CXCR4-6 | F:CACCGCTTCTACCCCAATGACTTG  R: AAACCAAGACATTGGGGTAGAAGC | 95 °C, 5 min; 95 °C to 85 °C, -2 °C/s; 85 to 25 °C, -0.1 °C/s |
| LentiCRISPR CXCR4-7 | F:CACCGTTCCAGTTTCAGCACATCA  R:AAACTGATGTGCTGAAACTGGAAC | 95 °C, 5 min; 95 °C to 85 °C, -2 °C/s; 85 to 25 °C, -0.1 °C/s |
| LentiCRISPR CXCR4-8 | F:CACCGAAGATGATGGAGTAGATGG  R: AAACCCATCTACTCCATCATCTTC | 95 °C, 5 min; 95 °C to 85 °C, -2 °C/s; 85 to 25 °C, -0.1 °C/s |
| LentiCRISPR CXCR4-9 | F:CACCGTAGCGGTCCAGACTGATGA  R:AAACTCATCAGTCTGGACCGCTAC | 95 °C, 5 min; 95 °C to 85 °C, -2 °C/s; 85 to 25 °C, -0.1 °C/s |
| LentiCRISPR CXCR4-10 | F:CACCGGATGAGGACACTGCTGTAG  R:AAACCTACAGCAGTGTCCTCATCC | 95 °C, 5 min; 95 °C to 85 °C, -2 °C/s; 85 to 25 °C, -0.1 °C/s |
| CXCR4 (homo) | F:TGGGCTCAGGGGACTATGACTCCATGAAGG  R:CAAACTCACACCCTTGCTTGATGATTTCCA | 94 °C, 30 s; 54 °C, 30 s; 72 °C, 45s; 28 cycles |
| CXCR4 (rhesus) | F:ATCTTCCTGCCCACCATCTACTCCA  R:CAAACTCACACCCTTGCTTGATGATTTCCA | 94 °C, 30 s; 54 °C, 30 s; 72 °C, 45s; 28 cycles |
| gag | F:ATCAATGAGGAAGCTGCAG  R:CACATAATCCACCTATC | 94 °C, 30 s; 54 °C, 30 s; 72 °C, 30s; 40 cycles |
| β-globin | F:ACACAACTGTGTTCACTAGC  R:TGGTCTCCTTAAACCTGTCTTG | 94 °C, 30 s; 54 °C, 30 s; 72 °C, 30s; 40 cycles |
| Off-target 1 | F:TTTGGGTTAATGAGTCAATGTGG  R:CAGAGCAAGGGTTCACCATTTCC | 94 °C, 30 s; 54 °C, 30 s; 72 °C, 30s; 28 cycles |
| Off-target 2 | F:ATGGGAAGGAGTGGTTCTAGGTT  R:GGGAGGTAAACTATCCTGGTCAA | 94 °C, 30 s; 54 °C, 30 s; 72 °C, 30s; 28 cycles |
| Off-target 3 | F：GTCCAGTGGAGCCAATAAAGGCTTG  R：TGAGGGTGATTGCTGAGGAGAACA | 94 °C, 30 s; 54 °C, 30 s; 72 °C, 30s; 28 cycles |
| Off-target 4 | F:CCAGGGTGAAGGAATGAGGACTG  R:GAAGTCGGGATGGTTGGCGTTAT | 94 °C, 30 s; 54 °C, 30 s; 72 °C, 30s; 28 cycles |
| Off-target 5 | F:AGCATTCCTGGCGTGGCAAACAC  R:GCAGGGCTTAATGGGACAAGTGG | 94 °C, 30 s; 54 °C, 30 s; 72 °C, 30s; 28 cycles |
| Off-target 6 | F：TTGGGTTTCCTCCAGGCTGTTAG  R：AAGGTCCCATAGCAAGTAGGAGGC | 94 °C, 30 s; 54 °C, 30 s; 72 °C, 30s; 28 cycles |
| Off-target 7 | F：CCCTCCACCCACATCCACATTCA  R：ATGAGATCTGGCTCCCATTGAAACA | 94 °C, 30 s; 54 °C, 30 s; 72 °C, 30s; 28 cycles |
